# Morphological and molecular characterization of *Henneguya cystigena* n. sp. (Cnidaria, Myxosporea) parasitizing the alimentary tract of yellowfin seabream, *Acanthopagrus latus*, in the East China Sea

**DOI:** 10.1051/parasite/2025048

**Published:** 2025-08-15

**Authors:** Bo Zhang, Fei Yin

**Affiliations:** 1 School of Marine Science, Ningbo University Ningbo 315832 Zhejiang Province PR China; 2 Key Laboratory of Aquacultural Biotechnology, (Ningbo University), Ministry of Education Ningbo 315832 Zhejiang Province PR China; 3 Key Laboratory of Green Mariculture (Co-construction by Ministry and Province), Ministry of Agriculture and Rural Ningbo 315832 Zhejiang Province PR China

**Keywords:** Myxozoa, Alimentary tract, SSU rDNA, Ultrastructure, Phylogeny

## Abstract

A novel myxosporean species was identified. The species formed spherical to ellipsoidal pseudocysts within the alimentary tract wall of a yellowfin seabream *Acanthopagrus latus* fished in the East China Sea. Histological examination confirmed that pseudocysts were localized within the submucosal layer of the stomach wall. Round to ellipsoidal myxospores exhibited two posterior caudal appendages, consistent with the morphological characteristics of the genus *Henneguya*. The myxospore body measured 9.6 ± 0.5 (8.6–10.6) μm in length, 7.3 ± 0.4 (6.8–7.9) μm in width, and 6.0 ± 0.2 (5.5–6.4) μm in thickness. Two equal pyriform polar capsules were observed, measuring 3.5 ± 0.3 (2.9–4.4) μm × 1.9 ± 0.2 (1.4–2.2) μm. Pairwise comparison referring to small subunit ribosomal DNA sequence revealed a highest identity of 94.19% with *Henneguya yokoyamai* Li *et al.*, 2012, supporting the classification of the specimens as a new species, *Henneguya cystigena* n. sp. Phylogenetic analyses demonstrated intermixed groupings of myxobolid species, highlighting persistent discrepancies between traditional morphological taxonomy and increasingly refined molecular phylogeny. To the best of our knowledge, this study represents the first description of a *Henneguya* species parasitizing a marine fish in the East China Sea near mainland China.

## Abstract (fr)

Une nouvelle espèce de Myxosporea a été identifiée, qui formait des pseudokystes sphériques à ellipsoïdaux dans la paroi du tube digestif d’un Pagre à nageoires jaunes, *Acanthopagrus latus*, pêché en mer de Chine orientale. L’examen histologique a confirmé la localisation des pseudokystes dans la couche sous-muqueuse de la paroi stomacale. Les myxospores rondes à ellipsoïdales présentaient deux appendices caudaux postérieurs, compatibles avec les caractéristiques morphologiques du genre *Henneguya*. Le corps de la myxospore mesurait 9,6 ± 0,5 (8,6–10,6) μm de longueur, 7,3 ± 0,4 (6,8–7,9) μm de largeur et 6,0 ± 0,2 (5,5–6,4) μm d’épaisseur. Deux capsules polaires piriformes égales ont été observées, mesurant 3,5 ± 0,3 (2,9–4,4) μm × 1,9 ± 0,2 (1,4–2,2) μm. La comparaison par paires des séquences de la petite sous-unité de l’ADN ribosomique a révélé que l’identité la plus élevée était 94,19% avec *Henneguya yokoyamai* Li *et al.*, 2012, ce qui soutient la classification des spécimens comme une nouvelle espèce, *Henneguya cystigena* n. sp. Les analyses phylogénétiques ont mis en évidence des regroupements mixtes d’espèces de Myxobolidae, soulignant des divergences persistantes entre la taxonomie morphologique traditionnelle et une phylogénie moléculaire de plus en plus affinée. À notre connaissance, cette étude constitue la première description d’une espèce d’*Henneguya* parasitant un poisson marin en mer de Chine orientale, près de la Chine continentale.

## Introduction

Myxosporea Bütschli, 1881 is a cosmopolitan group of cnidarian parasites (phylum Cnidaria Hatschek, 1888) that predominantly infect fish as vertebrate hosts [2, 32]. Within the group, the genus *Henneguya* Thélohan, 1892 belonging to family Myxobolidae Thélohan, 1892 represents one of the most speciose lineages, comprising over 250 described species primarily reported from freshwater ecosystems [13, 14, 28, 32]. Heavy infections by *Henneguya* spp. can exert deleterious effects on their hosts, with severe cases imposing survival pressure on both wild and cultured fish populations [24, 31, 34, 40].

China has the top fishery production worldwide and is a hot-spot area in documenting myxosporean diversity. Current documents indicate the presence of over 600 myxosporean species in Chinese waters, accounting for approximately 25% of known global diversity [6, 24, 30]. Despite this richness, taxonomic knowledge of *Henneguya* remains incomplete, with only about 35 species currently documented from China [6, 24, 40, 42]. While early descriptions relied heavily on morphological characteristics, contemporary studies increasingly incorporate molecular data to improve taxonomic resolution and reliability [7, 18].

The traditional discrimination between *Henneguya* and its relative *Myxobolus* Bütschli, 1882 has become increasingly problematic due to phylogenetic evidence demonstrating intermixed clustering patterns between the two genera [20, 24, 26]. These findings suggest that the essence of two caudal processes in *Henneguya* myxospores may represent a homoplastic character that has evolved independently multiple times. Notably, research efforts in China have predominately focused on those myxobolid species infecting freshwater fish [6, 40, 42], with marine representatives receiving comparatively less attention [24, 37].

The yellowfin seabream *Acanthopagrus latus* (Houttuyn, 1782) is an ecologically and economically important marine fish endemic to the East Asia Shelf [17]. To date, only three species of *Henneguya*, *i.e.*, *Henneguya lata* Chinh *et al.*, 2021 [7], *Henneguya yokoyamai* Li *et al.*, 2012 [22], and *Henneguya ogawai* Li *et al.*, 2012 [22], have been reported from this fish or its close relative Blackhead seabream *Acanthopagrus schlegelii.* In the present study, we document a novel *Henneguya* species forming distinctive pseudocysts in the alimentary tract wall of *A. latus* specimens collected from the East China Sea. Through integrated morphological, ultrastructural, histological, and molecular analyses, we provide comprehensive evidence supporting the taxonomic novelty of this parasite.

## Material and methods

### Ethics

Fish specimens were purchased dead. No further ethics statements are therefore required.

### Specimen collection and processing

Eight fish specimens of yellowfin seabream *Acanthopagrus latus* (Houttuyn, 1782) were purchased from native fisherman who capture marine fish in the East China Sea near the coast of Cangnan county (120°36′39″E, 27°23′36″N), Zhejiang province in November 2024. They measured 25 ± 4.7 (19–33.3) cm in whole length and 19.7 ± 3.6 (15.3–25.7) cm in body length. Following dissection, tissue samples from gills and abdominal organs (hepatopancreas, spleen, stomach, intestine, and kidney) were prepared for myxosporean examination under an Olympus CX33 microscope (Olympus Optical Co. Ltd., Tokyo, Japan). Photographs of fresh myxospores released from squashed pseudocysts were taken using a camera (Nanjing Eruoda Instrument Equipment Co. Ltd, Nanjing, China) mounted on the microscope and the images of 30 individuals were used to determine morphological parameters following the guidelines recommended by Lom and Arthur [27]. Measurements are given in micrometers unless stated otherwise.

### DNA extraction, amplification, and sequencing

The myxospores released from pseudocysts for the above-mentioned microscopic examination were fixed in 70% ethanol solution for subsequent molecular analysis. DNA was extracted from these myxospores using an E.Z.N.A. Tissue DNA Kit (Omega, Shanghai, China), following the manufacturer’s guidelines. Polymerase chain reactions (PCRs) were performed to amplify sequences of small subunit ribosomal DNA (SSU rDNA) in 25 μL reaction volumes containing 1 μL of genomic DNA, 1 μL of each primer at 10 μMol/L, 12 μL of 2× Es Taq MasterMix (Jiangsu Cowin Biotech Co., Ltd, Taizhou, China), and 10 μL of distilled water. The primer pairs ERIB1/ERIB10 [3] were used in PCR reactions. Cycling conditions were: pre-denaturation at 94 °C for 3 min; 35 cycles of denaturation at 94 °C for 30 s, annealing at 56 °C for 30 s, and elongation at 72 °C for 2 min; and terminal elongation at 72 °C for 5 min. PCR products were electrophoresed with 1% agarose gel in 1× Tris-Acetate-EDTA (TAE) buffer. The positive results were selected and commercially sequenced at Hangzhou Youkang Biotechnology Co., Ltd., China, with a self-designed primer Hen_F: 5′-ATAGAGCATGTGGTGGTTGG-3′ employed for sequencing process. Amplicons were assembled with the aid of DNASTAR software [4] and the assembled sequence was submitted to the GenBank database.

### Ultrastructural preparation

For scanning electron microscopy (SEM), myxospores pooled from the ruptured pseudocysts were dripped onto cell slides previously rinsed with 0.1 mg/mL Poly-L-Lysine Hydrobromide solution (Solarbio, Beijing, China). Then, the cell slides covered with myxospores were fixed in 2.5% glutaraldehyde (Ted Pella, Inc., Redding, CA, USA) in 0.1 M PBS (pH 7.4) at 4 °C for 24 h. Samples were then dehydrated through a gradient ethanol series (30%, 50%, 70%, 80%, 90%, 95%, 100%). After immersion into isopentyl acetate, myxospores were critical point dried in critical point using Hitachi HCP-2 Critical Point Dryer (Hitachi, Tokyo, Japan), coated with metallic gold using an Ion Sputter Coater MC1000 (Hitachi, Japan), and observed in an SU8600 Scanning Electron Microscope (Hitachi).

For transmission electron microscopy (TEM), the alimentary tract wall containing pseudocysts fixed in 2.5% glutaraldehyde solution were dehydrated following the process mentioned above. The infected tissues were infiltrated sequentially with: (1) a mixture of absolute acetone and a phenolic epoxy resin (2:1, v/v) for 12 h; (2) a mixture of absolute acetone and a phenolic epoxy resin (1:1, v/v) for 12 h; and (3) 100% phenolic epoxy resin for 24 h (all steps at 37 °C). An embedding process was performed using the same phenolic epoxy resin at 60 °C for 48 h. Ultrathin sections (70 nm) were cut in a Leica EM UC7 Ultramicrotome (Leica, Wetzlar, Germany) and mounted on formvar-coated copper 200-mesh grids. Later, ultrathin sections were then contrasted with 2% uranyl acetate and lead citrate, and dried before being observed with an HT7800 transmission electron microscope (Hitachi) running at 80 kV.

### Histopathological analysis

Tissue samples with pseudocysts were fixed in 4% paraformaldehyde solution for 48 h and then dehydrated in an ascending ethanol series. After embedding in paraffin, they were cut transversely about 5 μm in thickness, stained with hematoxylin-eosin (HE), and photographed using a camera (Nanjing Eruoda Instrument Equipment Co. Ltd) mounted on a CX33 microscope (Olympus Optical Co. Ltd.).

### Phylogenetic reconstruction

Phylogenetic relationships were inferred using the Bayesian inference (BI) and maximum likelihood (ML) methods. The SSU rDNA sequence of the isolate under study and 56 other sequences belonging to its closest relatives (identity of > 84%) according to BLASTn search (performed at February 28, 2025) were included in the dataset used for phylogenetic analyses. We selected SSU rDNA sequences of *Zschokkella nova* Klokacheva, 1914 [21] (DQ377690) and *Myxidium cuneiforme* Fujita, 1924 [15] (DQ377709) as outgroups. Sequences were aligned in the MAFFT v7.273 program using default parameters [19]. The conserved sites were processed in the online Gblocks 0.91b (http://phylogeny.lirmm.fr/phylo_cgi/one_task.cgi?task_type=gblocks) [5, 9, 10], with 1,323 bp for following analysis. Using the Bayesian information criterion (BIC), the program jModelTest 2.1.10 [8] was employed to determine the best optimal substitutional model (GTR + I + G) for phylogenetic analysis. Bayesian inference analysis was run in MrBayes v3.2.6 [33], with a chain length of 10^6^, frequency of a 100, and the first 25% of burn-in trees discarded. ML analysis was performed using IQTREE v1.6.12 [29] with the ultrarapid bootstrap method and 10^4^ bootstrap replicates [11]. The phylogenetic trees were annotated using Figtree v1.4.3. Additional annotations were retrieved from the corresponding literature and the taxonomy of the fish host primarily followed FishBase (https://www.fishbase.org/).

## Results

### Fish examination

**Figure 1:**
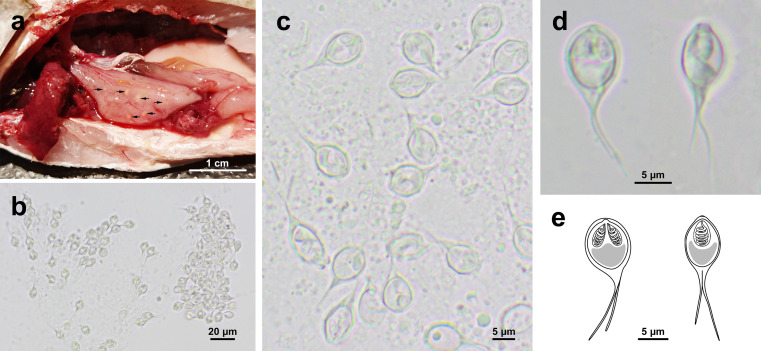
Photographs of fresh pseudocysts and myxospores of *Henneguya cystigena* n. sp. parasitizing the alimentary tract of *Acanthopagrus latus*. a: yellowish round to oval pseudocysts (arrows) scattered in the stomach wall; b–d: myxospores in frontal and lateral view; e: schematic drawing of a myxospore in frontal (left) and lateral (right) view.

Analyzed fish showed no gross abnormalities nor signs of disease. However, a dozen yellowish ellipsoidal pseudocysts were found scattered in the wall of the stomach and intestine of a single fish specimen (Figs. 1a). After pseudocyst rupture, round to ellipsoidal bivalve myxospores was observed, with a single caudal appendage extending from each shell valve under the microscope (Figs. 1b and 1c).

### Description of the new species

#### *Henneguya cystigena* n. sp. (Figs. 1–3)

urn:lsid:zoobank.org:act:0FD5F7FB-2F6A-446D-B3AB-CEB40DDC1972

Type host: Yellowfin seabream *Acanthopagrus latus* (Houttuyn, 1782).

Type locality: East China Sea near the coast of Cangnan county (120°36′39″E, 27°23′36″N), Zhejiang province, China.

Type material: Myxospores preserved in 80% ethanol, National Zoological Museum of China, Institute of Zoology, Chinese Academy of Sciences (IZCAS), collection number NBUECS241102 (syntypes). School of Marine Sciences, Ningbo University, collection number NBUECS241102-1 (syntypes).

Infection site: alimentary tract wall.

Prevalence: 12.5% (1 of 8 fish).

Etymology: the name “*cystigena*” derives from the generation (from the Latin *-gena*) of pseudocysts (from the Latin *cysti-*) by the present species.

**Morphology** (all measurements in μm): Myxospores 21.7 ± 2.2 (15.9–26.2) in total length. Myxospore body round to ellipsoidal, 9.6 ± 0.5 (8.6–10.6) in length, 7.3 ± 0.4 (6.8–7.9) in width, and 6.0 ± 0.2 (5.5–6.4) in thickness. Two equal-sized pyriform polar capsules located symmetrically in the anterior end of myxospores, measuring 3.5 ± 0.3 (2.9–4.4) × 1.9 ± 0.2 (1.4–2.2) (Figs. 1d–1e). Polar capsules placed at plane parallel to that of sutural line. Two caudal appendages, unequal in length, extending asymmetrically from posterior end, one per valve, one 13.4 ± 1.8 (9.7–16.6) in length, the other 10.2 ± 1.8 (6.4–13.3) in length.

#### Remarks

We compared phenotypic characters of the present species with those congeners of comparable morphological and morphometric data and parasitizing fish of close relatives (Table 1). All congeners, along with the present species, bear round or ellipsoidal myxospores, with the exception of *Henneguya latesi* whose myxospore is pyriform. *Henneguya lata* Chinh *et al.*, 2021 [7] was isolated from the same fish host, but its myxospore width and thickness, and length of polar capsules and caudal appendages are slightly smaller than those of the present species. *Henneguya yokoyamai* Li *et al.*, 2012 [22], *Henneguya ogawai* Li *et al.*, 2012 [22], *Henneguya cynoscioni* Dyková *et al.*, 2011 [12], *Henneguya latesi* Tripathi, 1952 [35], *Henneguya pagri* Yokoyama *et al.*, 2005 [38], and *Henneguya lateolabracis* Yokoyama, *et al.*, 2003 [39] possess longer myxospore length. Compared to the present species, myxospore width and thickness of *H. yokoyamai* and *H. ogawai* are smaller, but the measurement of their polar capsules is larger in dimension. Besides, *H. cynoscioni*, *H. pagri*, and *H. lateolabracis* have wider myxospores, but their dimensions of polar capsules are smaller than those of the present species. The caudal appendages of all comparable species here are equal in length, that is distinguishable from those of the present species. Additionally, the lengths of the caudal appendage of *H. lata* and *H. ogawai* are smaller and those of other congeners are larger than the present species. Accordingly, the present species differs from all the known congeners and represents a novel species. Therefore, we nominate it as *Henneguya cystigena* n. sp.

**Table 1:** Comparison of morphometric data of *Henneguya cystigena* n. sp. and its similar congeners.

Species name	TL	SL	SW	ST	PCL	PCW	LCA	CNPT	Infection site	Host	Location	References
*H. cystigena* n. sp.	21.7 ± 2.2 (15.9–26.2)	9.6 ± 0.5 (8.6–10.6)	7.3 ± 0.4 (6.8–7.9)	6.0 ± 0.2 (5.5–6.4)	3.5 ± 0.3 (2.9–4.4)	1.9 ± 0.2 (1.4–2.2)	13.4 ± 1.8 (9.7–16.6); 10.2 ± 1.8 (6.4–13.3)	3–4	alimentary tract wall	*Acanthopagrus latus*	China	The present study
*H. lata* Chinh *et al.*, 2021	19.3 ± 1.4 (16.5–21.5)	9.9 ± 0.5 (8.9–12.5)	6.7 ± 0.3 (6.1–7.6)	5.1 ± 0.2 (4.8–5.4)	3.2 ± 0.2 (2.8–3.9)	1.9 ± 0.2 (1.5–2.3)	10.0 ± 1.0 (8.3–11.6)	4–5	gills	*A. latus*	Vietnam	[7]
*H. yokoyamai* Li *et al.*, 2012	25.0 ± 1.7 (21.9–29.2)	11.0 ± 0.8 (10.1–13.7)	7.1 ± 0.4 (6.6–7.5)	5.6 ± 0.4 (4.5–6.4)	3.7 ± 0.4 (3.1–4.2)	2.0 ± 0.2 (1.8–2.4)	14.1 ± 1.6 (10.8–17.0)	–	gall bladder wall	*A. schlegelii*	Japan	[22]
*H. ogawai* Li *et al.*, 2012	21.1 ± 1.3 (19.2–23.4)	11.0 ± 0.8 (8.9–12.2)	6.9 ± 0.4 (6.3–7.5)	5.9 ± 0.4 (5.2–6.6)	4.3 ± 0.4 (3.8–5.2)	1.9 ± 0.2 (1.4–2.3)	10.0 ± 1.2 (8.4–12.7)	–	alimentary tract wall	*A. schlegelii*	Japan	[22]
*H. cynoscioni* Dyková *et al.*, 2011	38.6 (34.3–44.1)	10.4 (9.8–11.7)	8.8	5.8	3.3	2.0	28.0 (23.5–33.3)	2–4	bulbus arteriosus	*Cynoscion nebulosus*	USA	[12]
*H. latesi* Tripathi, 1952	–	9.0–10.8	6.3–8.2	5.4	3.6	2	17.2–25.4	–	gills, mouth cavity	*Lates calcarifer*	India	[35]
*H. pagri* Yokoyama, *et al.*, 2005	–	10.5 (9.9–11.9),	7.5 (6.4–8.4)	5.9 (5.4–6.4)	3.1 (2.5–4.0)	1.6 (1.5–2.0);	29.6 (24.8–34.7)	3	bulbus arteriosus	*Pagrus major*	Japan	[38]
*H. lateolabracis* Yokoyama, *et al.*, 2003	–	10.7 (9.9–11.9)	7.5 (6.4–7.8)	6.2 (5.9–6.4)	3.4 (3.0–4.0)	1.7 (1.5–2.0)	37.7 (30.7–49.5)	–	heart, gills	*Lateolabrax* sp.	Japan	[39]

Notes: TL: total length; SL: myxospore length; SW: myxospore width; ST: myxospore thickness; PCL: polar capsular length; PCW: polar capsular width; LCA: length of caudal appendage; CNPT: cycle number of polar tubules. All measurements are provided in micrometer (μm), dashes show no data.

### Molecular comparison

An SSU rDNA sequence comprising 1,932 bp was obtained, with accession number PV221478 available in GenBank. Based on BLASTn search, *Henneguya yokoyamai* infecting the gall bladder wall of *Acanthopagrus schlegelii* displayed highest similarity (94.19%, AB693053; Query Cover 100%) to that of the isolate under study. Comparatively, *Henneguya lata* (MT644624) from the gills of *A. latus*, and *Henneguya ogawai* Li *et al.*, 2012 [22] (AB693050) parasitizing the esophageal wall of *A. schlegelii* shared 93.72% (Query Cover 100%) and 93.15% (Query Cover 100%) molecular similarity, respectively.

### Electron microscopy

**Figure 2:**
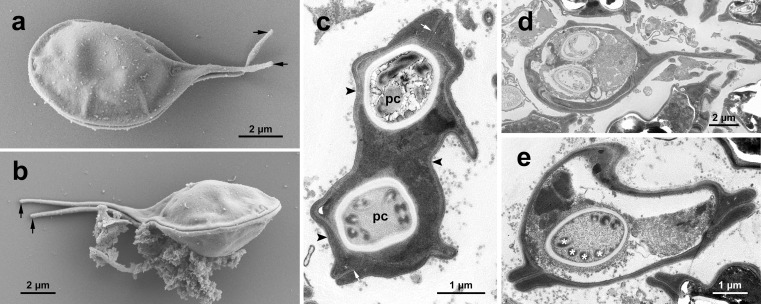
Scannning (a–b) and transmission electron microscopy (c–e) images of the myxospores of *Henneguya cystigena* n. sp. a–b: mxysopres exhibiting two smooth shell valves, with two tapered posterior cadual processes (black arrows). c: two polar capsules (PC) aligned parallel to the plane of sutural line (white arrows); shell valves electron-dense (black arrowheads); d: polar capsules positioned anteriorly, with a single caudal process extending posteriorly from the myxospore body. e: ultrathin transverse sections of a polar capsule revealing four coils of polar tubule (asterisks).

The observation of SEM showed myxospores exhibiting two smooth shell valves joined along a straight sutural line, with a caudal appendage extending from each valve and tapering posteriorly to the extremity (Figs. 2a and 2b). The results from TEM observation revealed an electron-dense myxospore valve (Figs. 2c–2e). The electron-dense sporoblast was observed inside shell valves, with two capsulogenic cells developing polar tubules. The longitudinal observation of myxospore reveals two polar capsules located anteriorly and one tapered caudal appendage at the posterior portion. Three to four turns of polar tubule cycled inside the polar capsule are observed in an ultra-sliced section.

### Histological analysis

**Figure 3:**
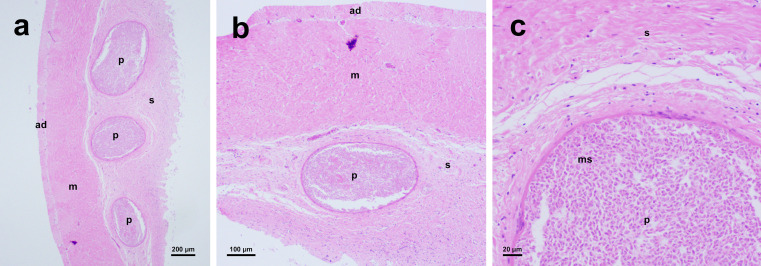
Histological sections of pseudocysts (p) formed by *Henneguya cystigena* n. sp. in the submucosa (s) of the host’s stomach segements (H & E). ad: adventitia; m: circular muscle layer; ms: myxospores.

Histological sections revealed round to ellipsoidal pseudocysts filled with myxospores developing in the submucous layer of the alimentary tract wall (Fig. 3a). Pseudocysts were entirely encapsulated by the connective tissue, with a peripheral edge confined by a delicate eosinophilic layer (Figs. 3b–3c). An inflammatory reaction was not observed in the surrounding tissue, but a slight distortion of circular muscle layer due to bulging of cysts could be observed. Myxospores inside pseudocysts were observed developing synchronously.

### Phylogenetic reconstruction

**Figure 4:**
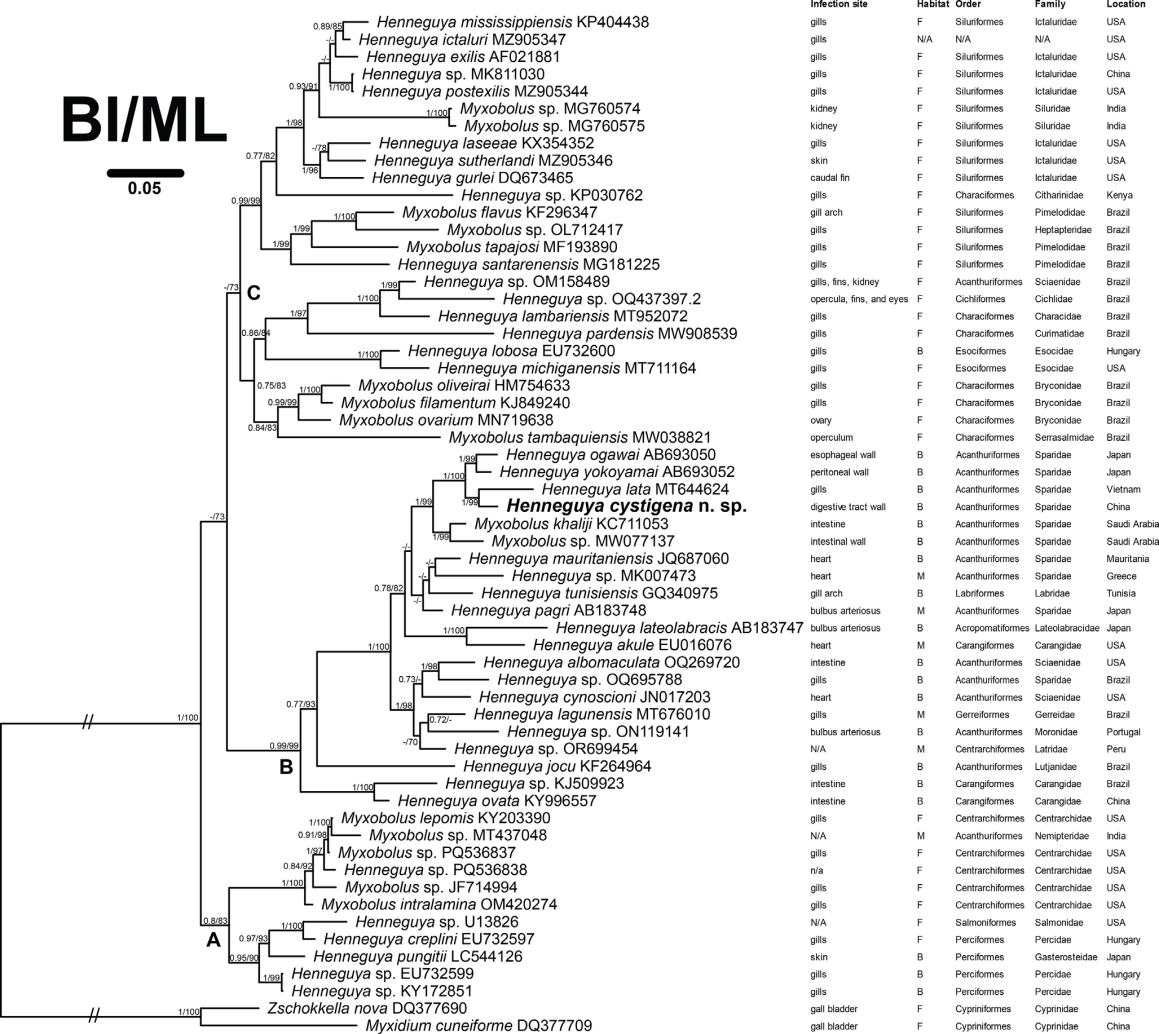
Phylogenetic tree reconstructed using Bayesian inference (BI) based on SSU rDNA sequences of *Henneguya cystigena* n. sp. and related myxosporeans. Bayesian posterior probability and maximum likelihood bootstrap values > 70 are indicated at each node. Dashes represent support values below 70. The species under study is highlighted in bold. For each organism, the following information is provided: GenBank accession number, infection site, habitat type (F: freshwatar; B: brackish water; M: marine; N/A: data not available), host affinity at order and family level, and host sampling location.

Trees obtained from BI and ML analyses exhibited almost consistent topologies (Fig. 4) and three main clades were artificially defined in the present work. Clade A at the basal position comprises myxosporeans primarily infecting fish belonging to Centrarchiformes and Perciformes. Clade B contains species that mostly parasitize marine fish, including those belonging to the family Sparidae (Eupercaria). *Henneguya cystigena* n. sp. clustering with *H. lata* formed a sister group to that composed of *H. ogawai* and *H. yokoyamai* in upper Clade B. Lastly, clade C comprises mainly species that infect Siluriformes, but also Characiformes and Esociformes. Species of *Henneguya* and *Myxobolus* are intermixed in all three clades.

## Discussion

The genus *Henneguya* represents one of the most speciose group within Myxosporea, exhibiting a global distribution and often displaying opportunistic pathogenicity in its fish host. In China, to the best of our knowledge, 35 species of *Henneguya* have been documented, predominantly from freshwater environments [6, 13, 24, 32, 40, 42]. Among these, *Henneguya latesa* Wu *et al.*, 1994 isolated from *Lates calcarifer* (Bloch, 1790) [37] and *Henneguya ovata* Liu *et al.*, 2018 detected in *Trachinotus ovatus* (Linnaeus, 1758) [24] are the only two species reported from marine habitats. Consequently, *Henneguya cystigena* n. sp. described herein represents the third *Henneguya* species identified along the coast of mainland China.

To ensure accurate species identification, we characterized the present species using a series of microscopic examinations. The observation of a pair of polar capsules lying on the plane parallel to the sutural line, coupled with a single caudal appendage extending from each shell valve, conforms to the taxonomic definition of the genus *Henneguya* [28]. While *Henneguya lata*, *H. ogawai*, and *H. yokoyamai* share *Acanthopagrus* fish hosts [7, 22], their myxospore dimensions differ from those of the present species. Furthermore, congeners exhibiting high morphometric resemblance are distinguished from the current isolate by divergence in at least one property, such as structure dimensions, infection site, or fish host specificity (Table 1).

The utilization of molecular data from SSU rDNA sequences significantly enhances accurate myxosporean identification and helps resolve taxonomic ambiguities. BLASTn analysis revealed a maximum sequence identity of 94.19% with all other SSU rDNA sequences available in the NCBI database. Although no universally specified threshold defines interspecific variability for myxosporeans, the observed 6% molecular divergence exceeds the approximately 1% difference commonly employed for delimiting species within Myxobolidae [7, 23, 40, 41]. Based on this substantial molecular distinction, in conjunction with morphological differences, we are confident in designating the present isolate as a novel species, named *Henneguya cystigena* n. sp.

The genus *Henneguya* is traditionally distinguished from *Myxobolus* by the presence of posterior caudal appendages [28]. However, in our phylogenetic reconstruction, *Henneguya* species appear intermixed with those of *Myxobolus* across all three main clades. Specifically, *H. cystigena* n. sp. forms a sister group to a cluster composed of *Myxobolus* species within clade B. This finding is consistent with previous studies [1, 16, 26] and supports the non-monophyletic origin of the genera *Myxobolus* and *Henneguya*, thereby challenging the reliability of caudal appendages as the sole diagnostic character for discriminating between these taxa. Additionally, the clustering patterns observed in our tree corroborate earlier phylogenetic analyses [1, 16, 18, 25, 26, 36, 41], providing further support for the coevolutionary history between these myxosporeans and their fish hosts. Signals related to habitat type, and infection site also exhibit some degree of congruence with grouping of the concerned species, consistent with previous findings [24, 26]. Notably, species within clade A and C primarily parasitize the fish body surface and gills, whereas those in clade B are mostly isolated from internal abdominal organs. This pattern suggests that ancestor of myxosporeans in clade B may have partially undergone an evolutionary adaptation from an original surface-infecting tropism to parasitizing internal organs.

## Conclusion

Combining morphological, ultrastructural, and molecular analysis, our results provide robust evidence for the description of a novel *Henneguya* species parasitizing the alimentary tract wall of wild yellowfin seabream *Acanthopagrus latus*. Phylogenetic analyses revealed intermixed clustering of myxobolid species, highlighting significant discrepancies between current morphological taxonomy and increasingly refined molecular phylogenies. Furthermore, this study expands our understanding of piscine parasite fauna in marine environments adjacent to mainland China.
